# Enhanced terahertz magneto-plasmonic effect enabled by epsilon-near-zero iron slot antennas

**DOI:** 10.1515/nanoph-2024-0665

**Published:** 2025-02-17

**Authors:** Hyoung-Taek Lee, Hoyeol Lee, Jeonghoon Kim, Miju Park, Changhee Sohn, Hyeong-Ryeol Park

**Affiliations:** Department of Physics, 131639 Ulsan National Institute of Science and Technology (UNIST), Ulsan 44919, Republic of Korea; Pohang Accelerator Laboratory, POSTECH, Pohang 37673, Republic of Korea

**Keywords:** terahertz metasurface, terahertz magneto-optics, epsilon-near-zero, Faraday rotation, gap plasmon effect, effective dielectric constant

## Abstract

Terahertz magneto-plasmonics plays a crucial role in platforms for isolation and sensing applications, operating at terahertz frequencies. In spite of recent efforts to enhance magneto-optic effects using metasurfaces, the mechanism for optimizing these effects remains unclear in the terahertz regime. Here we investigate terahertz magneto-optic effects using 100 nm-thick iron slot antennas with varying widths, ranging from 20 µm to 300 nm. Interestingly, as the width of slot antenna decreases, this enhancement peaks around 1 µm, after which the effect diminishes for smaller widths. Based on the effective medium theory, the slot antennas exhibit a maximum Faraday rotation angle near the epsilon-near-zero region. Although the field enhancements in the slot become stronger with the sub-micron widths, the magneto-optic effect may decrease with increasing effective dielectric constant due to gap plasmon effects in the sub-micron region. Our findings provide essential criteria for designing ferromagnetic metasurfaces with enhanced Faraday rotations at terahertz frequencies.

## 1 Introduction

Plasmonics enables the manipulation of light by structuring materials at the nanoscale to control light-matter interactions effectively [1], [2], [3]. In the terahertz (THz) regime, the resonant behaviors of slot antennas are explained by localized surface plasmons (LSPs) [4], [5], and the resonance of the slot antennas is determined by the shape of the slot antenna. Magneto-Optical (MO) effects, such as Faraday and Kerr rotations, could be enhanced by plasmonic structures, and this is so-called magneto-plasmonics [6], [7], [8], [9], [10], [11]. The MO effect enhanced by plasmonic structures has been utilized in applications such as optical isolation and sensing [12], [13], [14], [15], [16], [17]. The MO enhancement has largely been studied in the visible and near-infrared regions, where MO effects are typically driven by interband transitions [18], [19], [20], [21], [22]. However, ferromagnetic metals, unlike noble metals such as Au and Ag, often exhibit high losses for surface plasmon-polaritons in the visible range, which limits their ability to achieve strong enhancements [23], [24], [25], [26].

Enhanced MO effects are also required in the THz regime, where the needs for isolator systems and sensing platforms are similar to those in the visible spectrum [27], [28], [29], [30], [31]. In the THz range, the optical conductivity of ferromagnetic metals is typically comparable to that of noble metals, despite the fact that their optical conductivity is relatively low. As a result, ferromagnetic metals can be utilized to develop multifunctional plasmonic metasurfaces that incorporate both noble-metal-like performances and ferromagnetic properties. By structuring ferromagnetic materials to tailor their optical response to the external magnetic fields, one can enhance MO effects and facilitate effective light-matter interactions [23], [32], [33], [34], [35]. For example, a prior work has shown that circular hole patterned Co films enhanced Kerr rotation in the THz range, which is a reflective MO effect [36]. However, the mechanism in detail for maximizing THz magneto-plasmonic enhancements has not yet been fully understood despite these findings and substantial research efforts [27], [28], [37], [38].

In this study, we investigate how THz metasurfaces, such as slot antennas, patterned in ferromagnetic films influence enhancement of MO effects in the THz range. Specifically, we observe a transmission-type MO effect, Faraday rotation, through slot antennas patterned in an iron film. To realize this, iron slot antennas with varying its widths were fabricated using electron beam lithography and lift-off processes. The fabricated iron slot antennas were experimentally characterized by a transmission-type terahertz time-domain spectroscopy (THz-TDS) [39]. The effective medium of the ferromagnetic slot antennas can exhibit epsilon-near-zero (ENZ) properties, where the real part of the diagonal term of the dielectric function approaches zero near the peak position in the transmission spectrum. It has been demonstrated in the telecommunication wavelength range that MO effects can be significantly enhanced near this ENZ region [40]. In our results, the largest Faraday rotation angle was observed near the peak position in the spectrum, and interestingly, the angle increased as the width of the slot decreased, reaching a maximum at a 1 µm width, even though the Faraday rotation angle decreased when the width reduced to less than 1 μm. This phenomenon arises from the increased effective dielectric constant of the environments surrounding the slot antenna as the slot width decreases, enabled by the gap plasmon effect. Our results offer criteria for optimizing THz MO effects and provide important guidance in the design of magneto-plasmonic metasurfaces at THz frequencies.

## 2 Results and discussion

**Figure 1:**
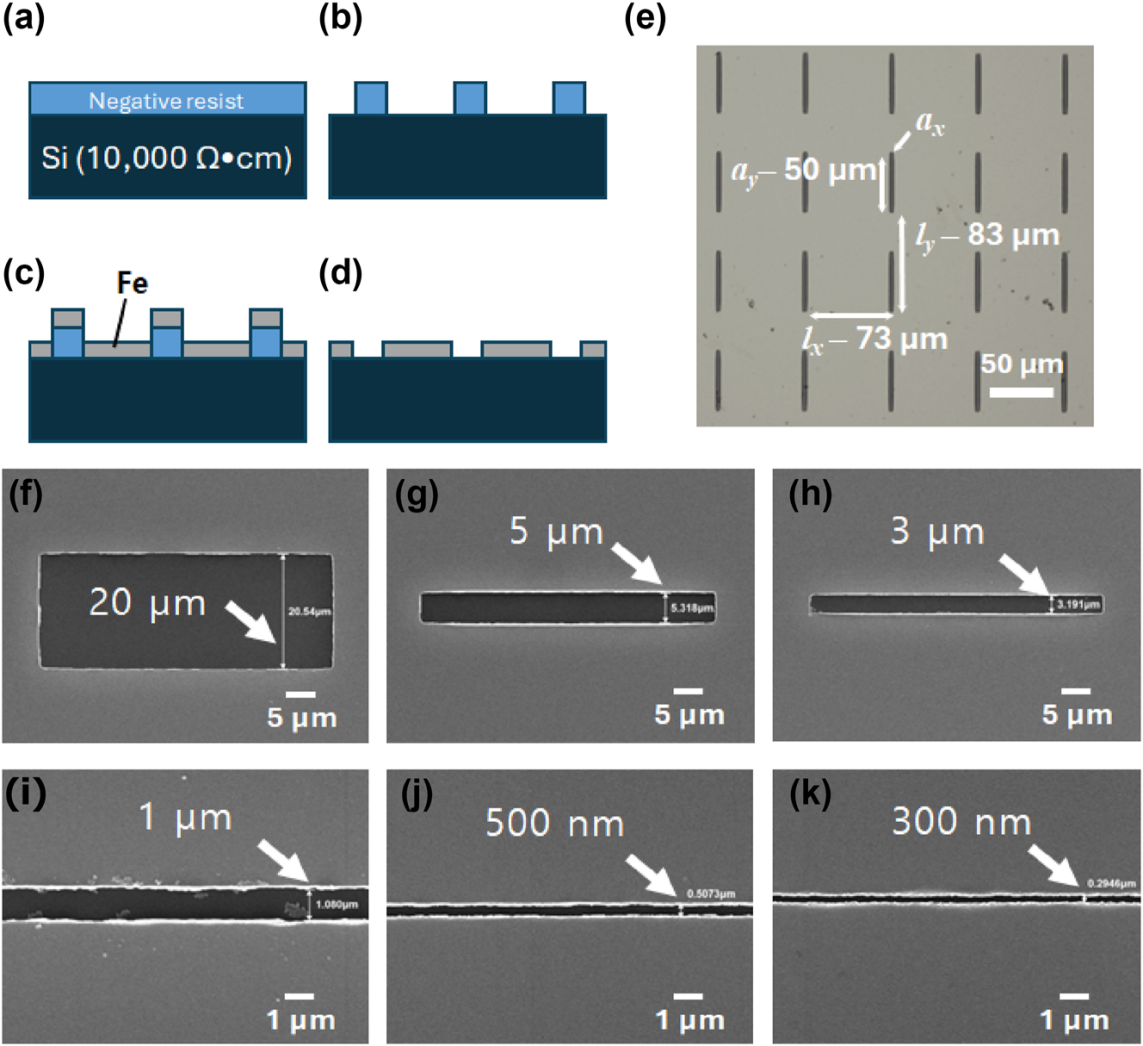
Fabrication of iron slot antennas using electron beam lithography. (a) A 500 nm-thick negative resist is spin-coated on Si substrate. (b) The resist pattern is completed through electron beam lithography and develop processes. (c) Fe 100 nm/Cr 5 nm is deposited using an electron beam evaporator. (d) The slot antennas were fabricated by removing the resist through a lift-off process. (e) The optical microscopy image of the slot antenna array. *a*_*x*_ is the width of slot, *a*_*y*_ is the length of slot, *l*_*x*_ is the period along the *x*-axis, and *l*_*y*_ is the period along the *y*-axis. (f–k) Scanning electron microscope (SEM) images of slot antenna patterns with the widths of (f) 20 µm, (g) 5 µm, (h) 3 µm, (i) 1 µm, (j) 500 nm, and (k) 300 nm, respectively.

As shown in Figure 1, the ferromagnetic Fe film was patterned with slot antenna arrays with the slot widths *a*_*x*_, ranging from 20 µm to 300 nm, the slot length *a*_*y*_ of 50 µm, the horizontal period *l*_*x*_ of 73 µm, and the vertical period *l*_*y*_ of 83 µm. The resonance frequency of the slot antenna primarily depends on the slot length *a*_*y*_ [41], [42], [43]. The structural parameters of the slot antenna were designed to obtain the maximum THz transmittance with the highest Q-factor at near 1 THz using an inverse design based on deep learning algorithms [44], in order to avoid overlap between the peak and dip positions on the transmittance spectrum. In Figure 1a–d, we have shown how the slot antennas were patterned using electron beam lithography, followed by the deposition of a 100 nm thick Fe film using an electron beam evaporator, and fabricated using lift-off process (see the details in the Methods section). In Figure 1e–k, scanning electron microscope (SEM) images of the slot antennas are presented, showing that the desired widths have been achieved. As shown in Supplementary material S1, the X-ray diffraction (XRD) analysis of the deposited bare iron film indicates that it is amorphous, and the magnetoresistance measurement reveals an out-of-plane coercive field of 84.4 mT.

When x-polarized light passes through a ferromagnetic material along the *z*-axis, it can experience Faraday rotation, causing y-polarization. In this case, the dielectric constant of the ferromagnetic material can be expressed in the form of a tensor with off-diagonal terms as follows:

$$\epsilon_{\text{Fe}}=\left(\begin{array}{ccc} \epsilon_{xx} & \epsilon_{xy} & 0\\ -\epsilon_{yx} & \epsilon_{yy} & 0\\ 0 & 0 & \epsilon_{zz} \end{array}\right)$$

where the diagonal terms of *ϵ*_*xx*_ = *ϵ*_*yy*_ = *ϵ*_*zz*_ are isotropic dielectric constant. When an external magnetic field is applied in the *z*-direction, the magneto-optical material exhibits an off-diagonal term, *ϵ*_*xy*_ = *ϵ*_*yx*_. It should be noted that the film remains structurally isotropic; however, the dielectric tensor is influenced by the MO effect induced by the external magnetic field. When an external magnetic field is not applied, the diagonal terms are maintained, but the off-diagonal terms become zero.

**Figure 2:**
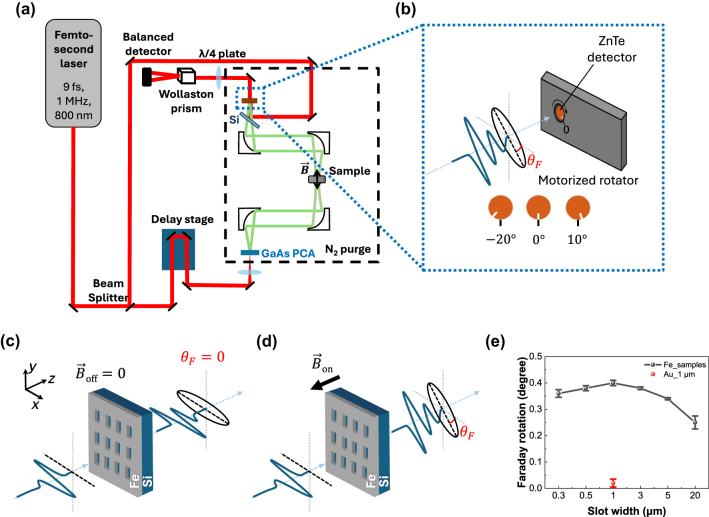
Experimental method for Faraday rotation measurement. (a) Schematic of a conventional THz-TDS setup. A femtosecond Ti:sapphire laser with a wavelength of 800 nm and up to 4 W output power excites a photoconductive antenna that generates THz pulses. (b) The THz pulse travels through the sample is detected by a ZnTe detector mounted on a motorized rotator. (c–d) Schematic of Faraday rotation effect by the iron slot antennas (c) without and (d) with an external magnetic field, where *θ*_*F*_ is the Faraday rotation angle. (e) Measured Faraday rotation angles of the iron (Fe) slot antennas with the varying widths between 0.3 and 20 μm are compared with the Faraday rotation of gold (Au) slot antennas with the width of 1 μm.

In Figure 2a, we performed transmission-type THz-TDS to determine how much the polarization of transmitted light through the iron slot antennas rotated compared to the incident x-polarized light. Especially, the detector crystal of ZnTe was mounted on a motorized rotator to directly measure its polarization rotation [45], [46], [47]. Before mounting the sample, the photo-conductive antenna and the ZnTe detector were aligned to the maximum at 0°, and then the sample was mounted and aligned precisely. After that, each sample was measured by rotating the motorized rotator with detection crystal between −20 and +10° with intervals of 2°. As a result of comparing the peak amplitude at each angle with and without an external magnetic field, the Faraday rotation angle was determined (see the details in the Faraday rotation analysis of Supplementary material S2). The sample was placed on a 3 mm × 3 mm aperture, and an out-of-plane magnetic field of approximately 90 mT was applied using a donut-shaped neodymium magnet (Figures 2c and d).

The measured Faraday rotation angles, as shown in Figure 2e and Table 1, demonstrate the *a*_*x*_-dependent enhancement of the MO effect. As the slot width decreases, the Faraday rotation continues to increase until the width reaches 1 µm, demonstrating significant improvement. The Faraday rotation angle is enhanced by approximately 160 % when comparing the results for the width of 1 µm to the width of 20 µm. This enhancement may be due to the strong light-matter interaction confined within sub-wavelength structures [48], [49], [50]. It is interesting, however, to note that the enhancement tends to decrease as slot widths decrease below 1 μm. This behavior illustrates that while reducing the gap size up to a certain threshold improves MO response, going below that threshold (especially down to the nanometer scale) introduces additional physical phenomena that can hinder performance, including strong plasmonic absorption or field localization that confines energy too tightly.

**Table 1:** A table of the Faraday rotation angles measured for the Fe slot antennas with the varying widths between 0.3 and 20 μm and the Au slot antennas with the width of 1 μm.

Samples	Faraday rotation angle
Au, *a*_*x*_ = 1 μm	0.02°
Fe, *a*_*x*_ = 20 μm	0.25°
Fe, *a*_*x*_ = 5 μm	0.34°
Fe, *a*_*x*_ = 2 μm	0.38°
Fe, *a*_*x*_ = 1 μm	0.4°
Fe, *a*_*x*_ = 0.5 μm	0.38°
Fe, *a*_*x*_ = 0.3 μm	0.36°

The gold slot antenna at *a*_*x*_ = 1 μm exhibited a Faraday rotation angle close to zero within the margin of error, which was about 20 times smaller than that of the patterned Fe film samples. Furthermore, when we compare the unpatterned Fe film with the patterned Fe film, the iron exhibits extremely high conductivity in the THz range, resulting in a transmitted electric field amplitude of less than 1 % even for thin films of 50 nm [51]. In such cases, the transmitted amplitude is too low to distinguish differences in angle dependence. The experimental results in Supplementary material S3 contain the measured peak amplitudes for each sample with all rotation angles of the ZnTe detector, and it demonstrates the challenges in determining the Faraday rotation angle in the bare Fe film. It should be noted that as a result of patterning the bare Fe film, it will be possible to measure the Faraday rotation angle, which was difficult to measure with a transmission-type THz setup.

**Figure 3:**
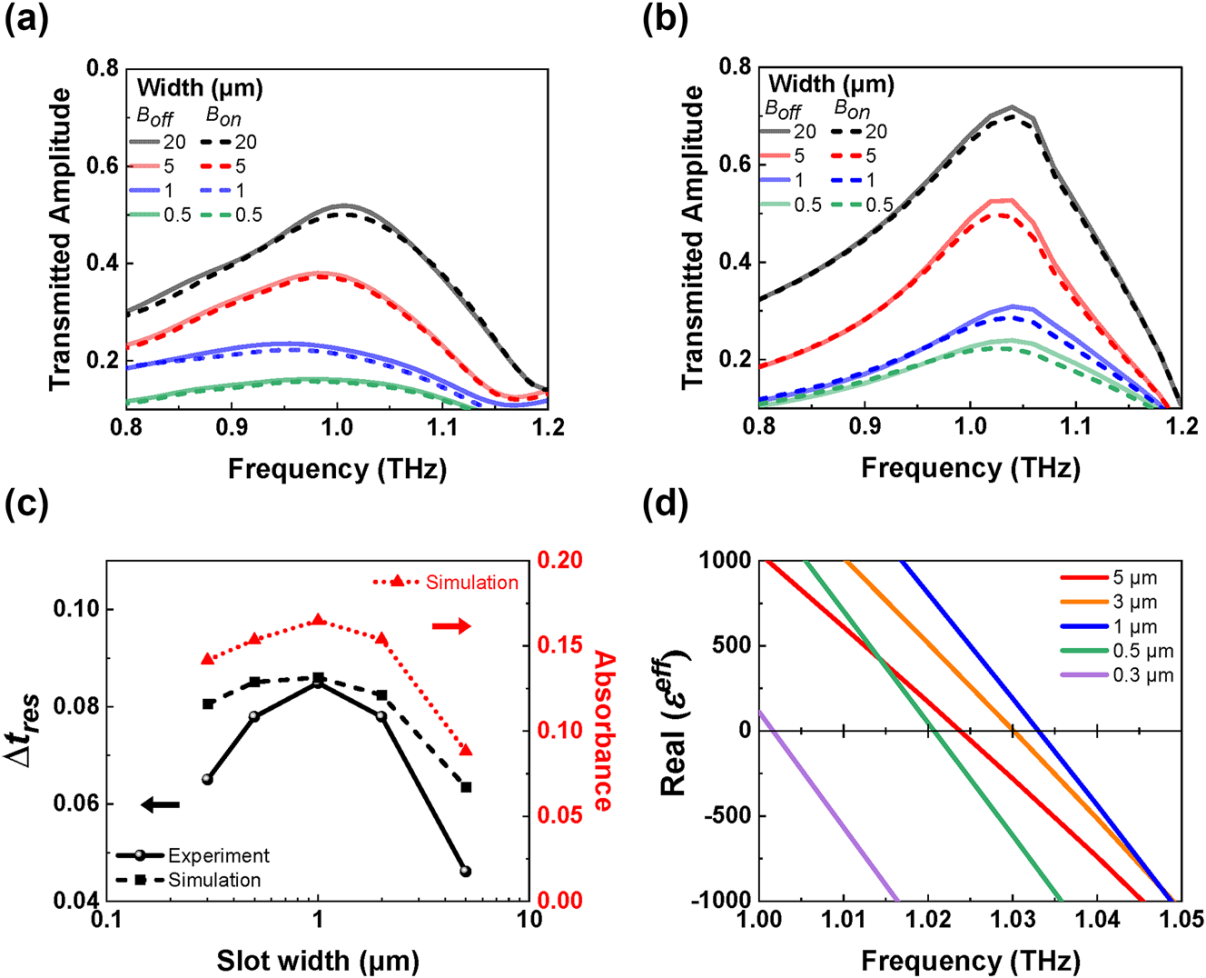
Transmitted amplitude spectra and magneto-optical enhancement of Fe slot antennas. (a) Measured and (b) simulated transmitted amplitude spectra of the Fe slot antennas with the varying slot widths when the magnetic field is on and off ( ${\vec{B}}_{\mathrm{on}}$ and ${\vec{B}}_{\mathrm{off}}$ ). (c) The normalized peak amplitude difference, $\Delta t_{res}=\left|t_{res}^{on}-t_{res}^{o\mathrm{ff}}\right|/t_{res}^{o\mathrm{ff}}$ , and the absorbance as a function of slot width. Here, $t_{res}^{on}$ and $t_{res}^{o\mathrm{ff}}$ are the resonant peak amplitudes with and without the external magnetic field, respectively. The absorbance is calculated using the formula 1 − ***T*** − ***R***, where ***T*** and ***R*** represent the transmittance and the reflectance without the external magnetic field. (d) The real part of the dielectric constants of the effective medium $\left(\varepsilon^{\mathrm{eff}}\right)$ with the varying widths as a function of frequency. It reveals the occurrence of an epsilon-near-zero (ENZ) region near the peak position.

For a more detailed understanding of the slot width-dependent Faraday rotation angles, we compared transmitted amplitude spectra of the Fe slot antennas with varying widths when the magnetic field is on and off, using the THz-TDS experiments (Figure 3a). To verify the MO effects induced by the off-diagonal term in the dielectric function of ferromagnetic Fe film, we reproduced the MO effect with and without the magnetic field using full-wave optical simulations, as shown in Figure 3b (see the details of the numerical simulation in Methods) [52]. The simulation results are in good qualitative agreement with the experimental results. The observed resonance behavior and enhancement of MO effects can be attributed to LSPs, that cause the charge distribution and current flow within the slot antenna. The slight difference between transmitted amplitudes in the experiments (Figure 3a) and the simulations (Figure 3b) is attributed to stitching errors during the e-beam lithography process. Due to the diffraction limit of UV light, nanometer-scale patterning is difficult to achieve using photolithography. A large area of 5 mm × 5 mm is patterned using e-beam lithography, which can pattern only 400 µm × 400 μm at one position of the sample stage. This stitching error that occurs when moving from one position to the neighbor position, leads to broader spectral features and lower peak amplitude (see the details of stitching errors in Supplementary material S4).

Figure 3c illustrates the maximum changes due to switching on and off the magnetic field for each sample, with the largest difference near the peak position. Furthermore, the MO effect has been enhanced as the slot width decreases down to 1 µm. Interestingly, however, we observed that with even smaller widths of a few hundred nanometers, the enhancement of the Δ*t*_res_ diminishes. The reason why the Δ*t*_res_ is maximized at 1 µm width will be discussed later. In Figure 3c, the experimentally observed Δ*t*_res_ of approximately 8 % at 1 µm sample requires an angle of $\theta ={cos}^{-1}\left(0.92\right)=23^{◦}$ . This is significantly larger than the relatively small Faraday rotation angle of 0.4°. When the dielectric tensor includes the off-diagonal term, it can influence the diagonal terms of the refractive index. As a result, Δ*t*_res_ observed is greater than what would be expected solely based on the Faraday rotation angle. The absorbance of the slot antenna in Figure 3c was obtained through simulations under zero magnetic field (For the details of absorbance and absorption coefficient spectra, see the Supplementary material S5). Interestingly, the absorbance reaches its maximum value at the slot width of 1 μm, due to plasmonic absorption by the slot antenna that results in a giant Faraday rotation effect [19], [53].

Using the effective medium theory to model the Fe slot antennas as a single film, the dielectric constant of the effective medium can be derived by analyzing the transmitted amplitude and phase information from the simulations. Details on extracting the dielectric constant from the effective medium (*ϵ* ^eff^) are provided in Supplementary material S6, with Figure 3d showing the real part of *ϵ* ^eff^ as a function of frequency. It is noted that the real part of the *ϵ* ^eff^ approaches zero near the peak position. An earlier work [40] also showed that MO effects are most significantly enhanced in the vicinity of the ENZ region. Furthermore, the ENZ region of the effective medium at the width of 1 µm appears at the highest frequency, which might explain why the MO effect has been maximized with the 1 µm width slot.

**Figure 4:**
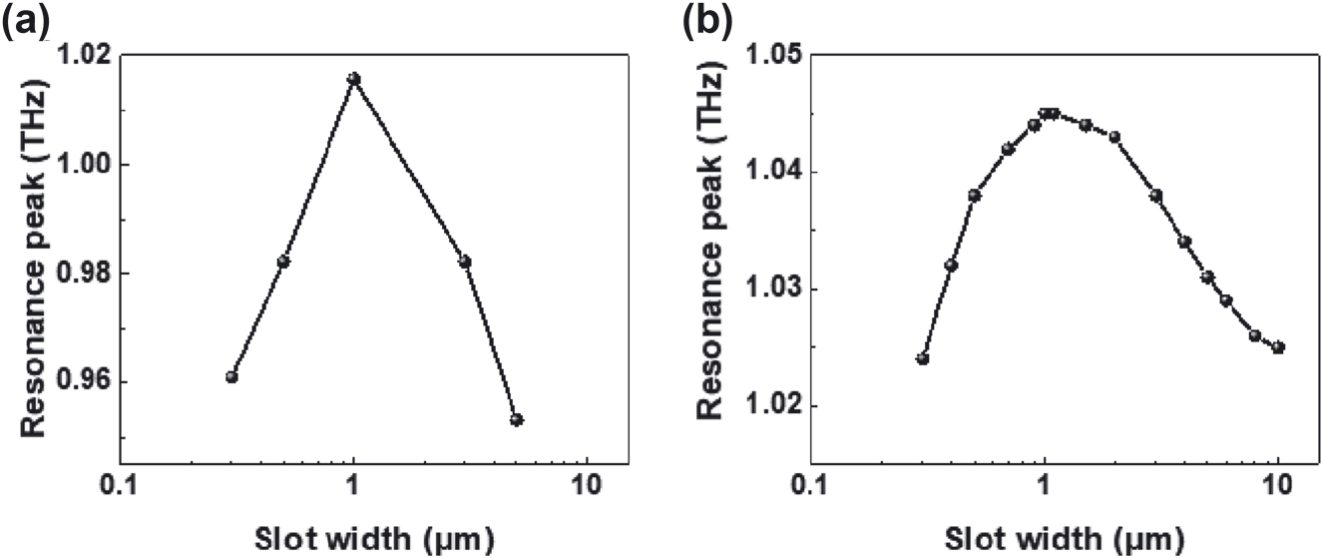
Resonance peak positions as a function of the slot width are shown in (a) the experiments and (b) the simulations.

Due to the unintuitive behavior appearing at the hundred nanometers widths, we focus on the peak position as it varies with the width of the iron slot antenna when no magnetic field is applied. Here, the slot antenna is not considered as an effective medium. Instead, we analyze it from the perspective of the effective index of the surrounding materials, including air and the substrate. It is important to note that, according to antenna theory, the peak position is primarily determined by the vertical length of the rectangular hole, *a*_*y*_ [41], [42]. However, small variations in the effective index surrounding the slot antenna due to the width, *a*_*x*_, can also induce changes in the peak position. According to the experimental results in Figure 4a, for samples wider than 1 µm, a blue shift occurs as the width decreases, whereas for samples narrower than 1 µm, a red shift is observed as the width decreases. The simulation results for more slot widths in Figure 4b show a similar shift in peak position. The dielectric constant of the material surrounding the slot antenna influences the resonant feature of the antenna [41]. When the slot width is even smaller than the wavelength, the gap plasmon effect will become non-negligible, causing the resonance peak shift [54], [55]. Actually, in the widths between 1 and 10 µm, the MO effect continues to be enhanced, while the decreasing effective index causes a blue shift in the peak position. Interestingly, the peak shift changes to a red shift at below 1 µm where the gap plasmon effect occurs, and the MO effect diminishes. Based on our findings, we conclude that when the effective dielectric constant of the environmental material surrounding the slot antenna is lowest, the Faraday rotation angle is enhanced the most.

## 3 Conclusions

We experimentally demonstrate that the magneto-plasmonic effect of iron slot antennas can be significantly enhanced through precise structural manipulation of ferromagnetic iron films at the THz region. By varying slot widths and applying an external magnetic field, we achieved an approximately 160 % increase in the Faraday rotation angle with the slot width of 1 µm compared to larger gaps. This finding emphasizes the potential of structural manipulation to improve magneto-plasmonics performance effectively. An effective medium analysis revealed that the peak magneto-plasmonics response occurred near the ENZ region, which is critical when considering magneto-plasmonics enhancements. Under magnetic field on/off conditions, both experimental and simulation results showed that the enhancement of the magneto-plasmonic effect was most pronounced at the width of 1 µm. Further reduction below this threshold, however, presented limitations, possibly resulting from an increase in the effective dielectric constant of the environmental materials surrounding the slot due to the gap plasmon effect. Our findings demonstrate that optimizing structural parameters, particularly the effective dielectric constant within the slot, plays a critical role in maximizing the magneto-plasmonic effect. This work highlights the potential for tailored ferromagnetic film patterning to advance magneto-plasmonic applications in the THz regime, offering insights for further optimization.

## 4 Methods

**Fabrications**. The ferromagnetic Fe slot antennas were fabricated using electron beam lithography. First, a negative electron beam resist (MA-N 2405) is spin-coated with a 500 nm thickness on a 500 µm-thick undoped silicon substrate. After spin-coating, samples are baked on the hot plate at 90 °C for 1 min 20 s. As the next step, the electron beam is illuminated on the electron beam resist at 100 μC/cm^2^ at an acceleration voltage of 30 keV. Subsequently, the 100 nm iron film was deposited with 5 nm Cr adhesion layer on the samples using electron beam evaporation. Finally, the lift-off process is performed to remove the electron beam resist, and the Fe slot antenna arrays are completed.

**Simulations**. The full-wave optical simulations were performed to analyze the transmitted amplitude spectra of the Fe slot antennas using the finite element method (COMSOL Multiphysics, wave optics module). The Fe slot antennas with a thickness of 100 nm on a silicon substrate has the following parameters: horizontal length (or slot width) of rectangular hole of *a*_*x*_: 0.3–20 µm, vertical length of rectangular hole of *a*_*y*_: 50 µm, x-periodicity of *l*_*x*_: 73 µm, and y-periodicity of *l*_*y*_ at 83 µm. The refractive index of the air inside and above the slot is 1.0, while the refractive index of the silicon substrate below the slot is 3.4. The diagonal term of the dielectric function of the Fe film was obtained using the Drude model [56]. as follows:

$${\tilde{\epsilon }}_{xx}\left(\omega \right)={\tilde{\epsilon }}_{yy}\left(\omega \right)={\tilde{\epsilon }}_{zz}\left(\omega \right)=\epsilon_{\infty }-\frac{\omega_{p}^{2}}{\omega^{2}+i\omega \omega_{\tau }}$$

where *ω*_*p*_ = 0.622 × 10^4^ THz and *ω*_*τ*_ = 27.7 THz are the Drude parameters of a bulk iron [56]. When the external magnetic field is applied to the iron slot antennas, we used the off-diagonal terms of the following fitting parameters: *ϵ*_*xy*_ = *ϵ*_*yx*_ = 0.45*i* ⋅*ϵ*_*xx*_, which was selected based on our experimental results. The perfectly matched layers were applied to the top and bottom of the simulation region to prevent unwanted reflections. Periodic boundary conditions were applied to the *xz*- and *yz*-boundaries. To process the significant computational load due to the thin thickness of the pattern, a quarter geometry was used, and adaptive meshing was applied.

## Supplementary Material

Supplementary Material Details
