# Supplementary material for: Enhanced terahertz magneto-plasmonic effect enabled by epsilon-near-zero iron slot antennas
Source: Nanophotonics. 2025 Feb 17;14(8):1257–64. doi: 10.1515/nanoph-2024-0665 (PMC12019947; doi:10.1515/nanoph-2024-0665)
Supplement: Supplementary file 1 — Supplementary Material Details [file j_nanoph-2024-0665_suppl_001.pdf]

Supplementary Material

Enhanced terahertz magneto-plasmonic effect enabled by epsilon-near-zero iron slot antennas

Hyoung-Taek Lee[1,2†], Hoyeol Lee[1,†], Jeonghoon Kim[1], Miju Park[1], Changhee Sohn[1], Hyeong-Ryeol Park[1,*]

[1] Department of physics, Ulsan National Institute of Science and Technology (UNIST), Ulsan 44919, Republic of Korea

[2] Pohang Accelerator Laboratory, POSTECH, Pohang 37673, Republic of Korea

[†] These authors contributed equally to this work.

[*]Corresponding author:

Hyeong-Ryeol Park, nano@unist.ac.kr

S1. XRD and saturation magnetic field for 100 nm-thick iron film

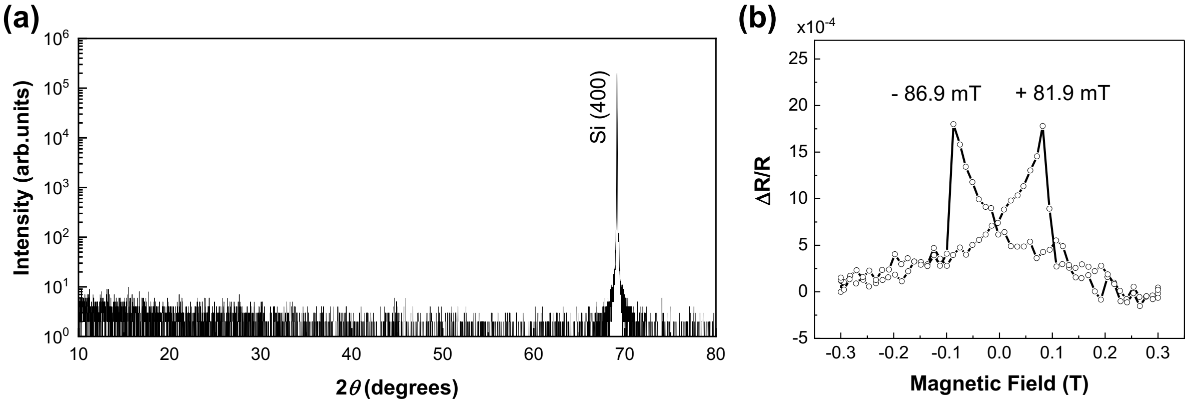

Figure S1. (a) X-ray diffraction (XRD) θ-2θ scan of a 100 nm-thick Fe film. The substrate Si (400) peak is sharply visible, while the absence of Fe peak indicates that the film is deposited in an amorphous state with no crystalline structure. High-resolution X-ray diffraction was conducted using the D8 Advance High-Resolution X-ray Diffraction (Bruker) with Cu K-α1 wavelength to characterize the non-crystalline iron film. A 0-D Lynxeye detector is used in θ-2θ scan, which has 0.01° increment with a scan speed of 0.5 s per step for a range from 10° to 80°. (b) Magnetoresistance measurements of a 100 nm-thick iron film under out-of-plane magnetic fields at 300 K. The peaks correspond to the coercive field, averaging 84.4 mT. The magneto-transport measurement was performed by using the Cryogen-Free Measurement System (Cryogenic Ltd). The resistance was measured in van-der Pauw configuration at 300 K under an external out-of-plane magnetic field -300 mT to +300 mT at a 50 mT/min rate. To prevent the crystallization of the amorphous thin film, all electrode contacts were made using silver paste, which was cured at room temperature.

S2. Faraday rotation analysis using terahertz time-domain spectroscopy

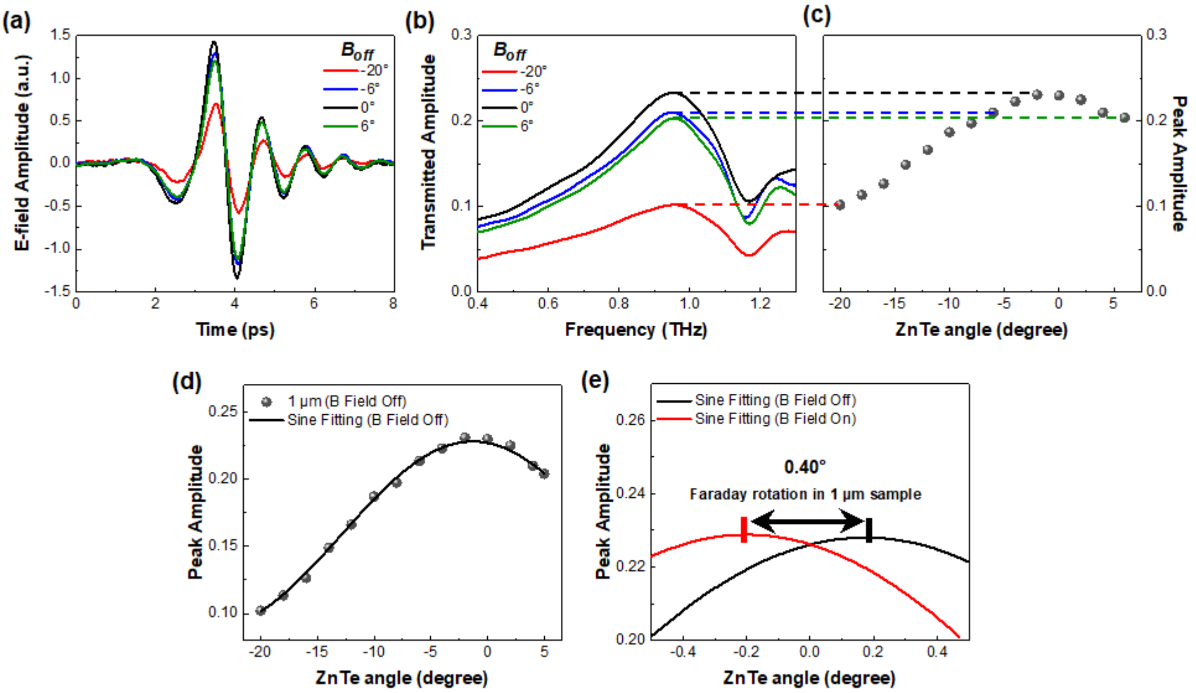

Figure S2. (a) Time data obtained by rotating the ZnTe mounted on a motorized rotator. (b) Transmitted amplitude spectra on frequency domain obtained by Fourier-transforming the time data at each angle. (c) The resonance peaks of the frequency spectra were plotted as a function of the ZnTe angle. (d) The resonance peak amplitude data without magnetic field (B_{off}) were fitted to a sine function (e) Sine-fitting data of the 1 μm-width slot antennas were obtained by repeating the processes of (a)-(c) under the magnetic field (B_{on}), and the shift in the sine graph was measured.

Figure S2(a) shows the time-domain data of the terahertz electric field transmitted through the iron slot antennas with the width of 1 μm obtained by terahertz time-domain spectroscopy (THz-TDS) [1]. The time-domain data are acquired by measuring the electric field of the THz pulse as a function of time. To convert this time-domain data into frequency-domain information, the Fast Fourier Transform (FFT) is typically applied [2]. As shown in Figure S2(b), this process decomposes the time-dependent signal into its frequency components, resulting in a frequency-domain spectrum. The resonance peak amplitudes in the frequency spectra at each angle (Figure S2(c)) were fitted to a sine function, as shown in Figure S2(d). When the polarization of light is rotated due to the magneto-optical effect, the detector will measure the maximum resonance peak at an angle different from the angle at which the maximum resonance peak appears in the absence of a magnetic field [3]. This rotation corresponds to the Faraday rotation angle. To determine this, an external magnetic field of 90 mT is applied to the sample, and the procedure outlined in Figure S2(a-d) is repeated. Figure S2(e) shows that the Faraday rotation angle is determined by comparing the shifted sine fitting graphs with and without the magnetic field.

S3. Experimental results

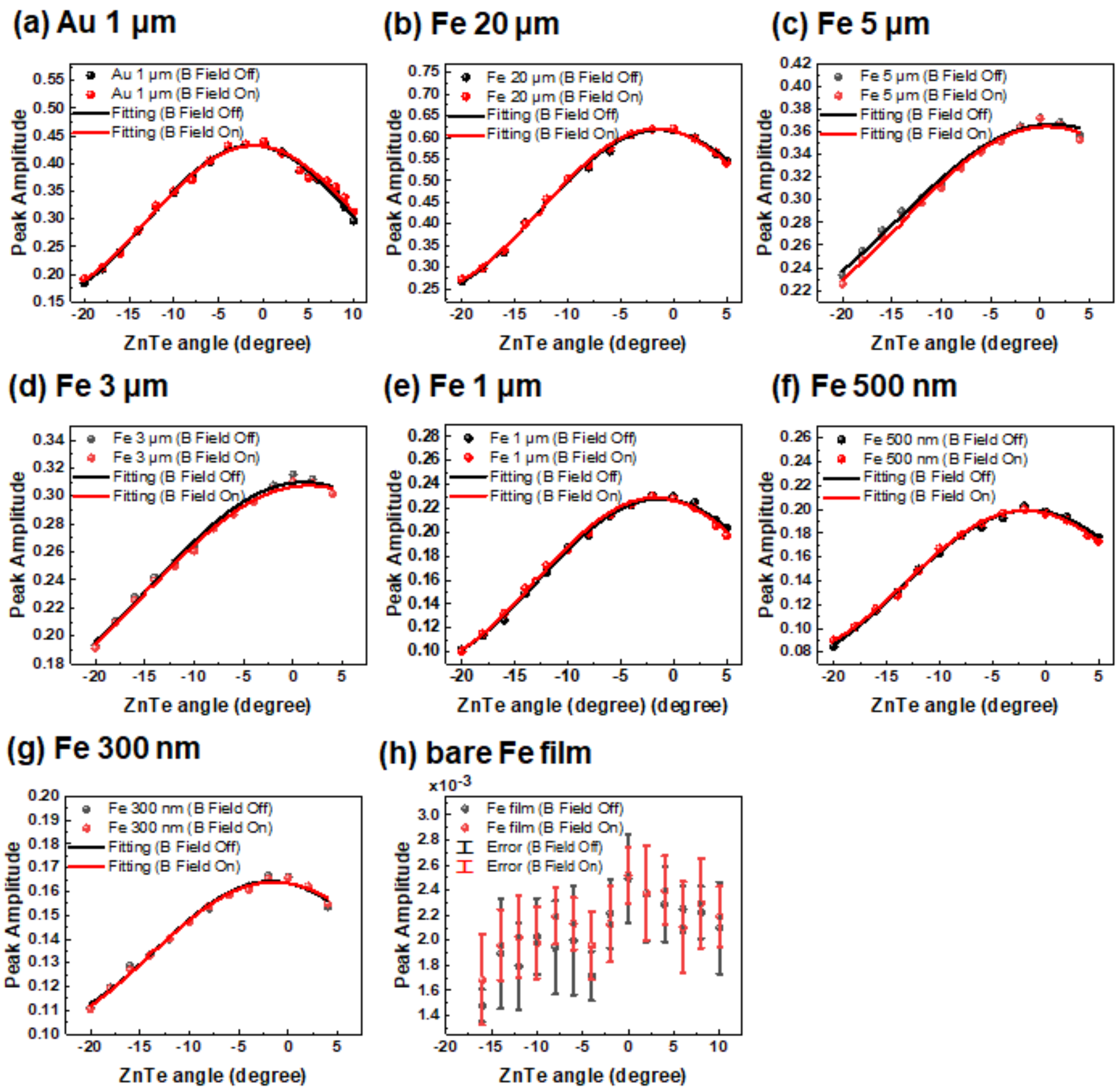

Figure S3. (a-h) Resonant peak amplitude and sine fitting graphs for each angle of the samples with and without the external magnetic field. Graphs of (a) 1 μm-width slots with a 100 nm thick gold film, and (b) 20 μm-, (c) 5 μm-, (d) 3 μm-, (e) 1 μm-, (f) 500 nm-, and (g) 300 nm-width slots with 100 nm thick Fe films, respectively, are shown. (h) A graph of bare Fe film with the same thickness of 100 nm is shown with the error-bars.

S4. Stitching error during e-beam lithography process

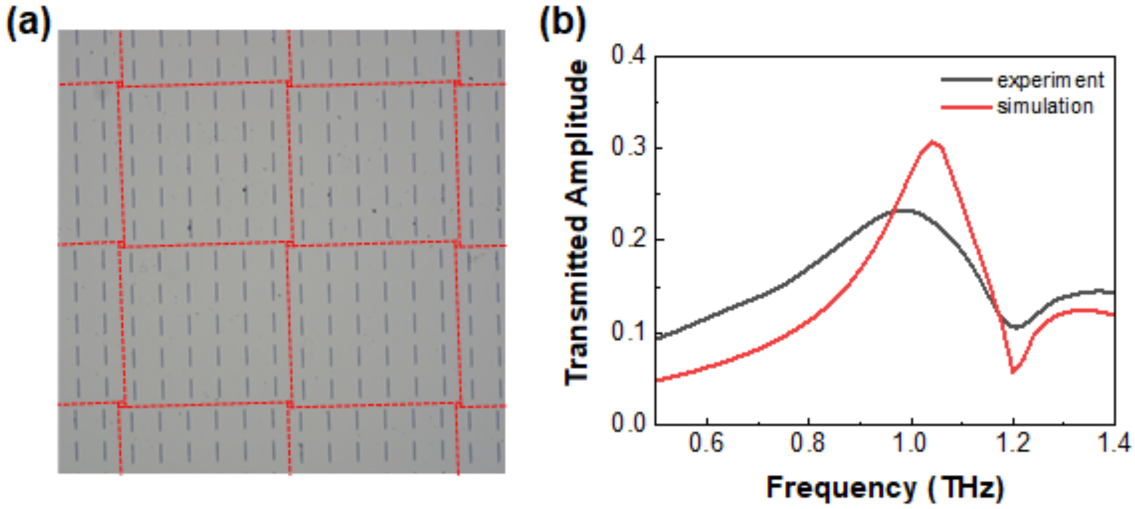

Figure S4. (a) An optical microscope image of the sample showed a distortion of 4.5 µm at the edges of the array due to stitching error. (b) The transmitted peak amplitude in the simulation for the 1 µm-width slot antennas is 0.31, while the experimental data shows a value of 0.23.

In this work, the slot antenna array pattern was fabricated by electron beam lithography using the FE-SEM [4]. Due to the limited area of the electron beam, the equipment could not pattern the entire 5 mm × 5 mm sample area at one position. Instead, the stage was moved incrementally to pattern 400 µm x 400 µm sections, and the pattern was completed over 144 times for a total of 20 minutes. However, in this process, Misalignments at the edges of the writing field such as in Figure S4(a) occurred due to stitching errors caused by the limited precision of the stage and the constraints of the electron beam position control system [5]. The fabricated samples exhibited a misalignment of approximately 4.5 µm for the 400 µm x 400 µm corners. The error rate of the fabricated samples compared to the data used for the simulation was 1.13%. In this case, a misalignment of 6.16% occurred between the antennas at the corners. Resonance peaks and dips are not significantly affected by this stitching error, but the overall transmitted amplitude may be affected [5]. Figure S4(b) shows the simulated data and experimental data of the 1 µm width slot sample. There was a 26% discrepancy between the frequency-domain data from the simulation and the experiment. Photolithography can be used for micrometer-scale slots, but electron beam lithography was also employed for micrometer-scale slots in order to minimize quality differences between samples, especially in larger patterns due to stitching errors.

S5. Absorbance and absorption coefficient spectra of the slot antennas

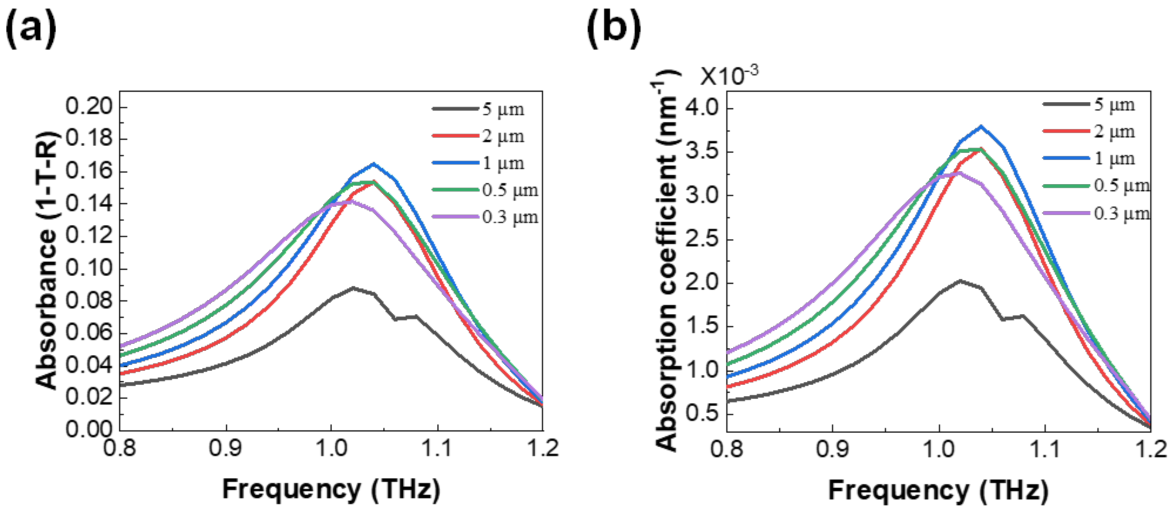

Figure S5. (a) The absorbance spectra for the slot antennas with varying the slot widths have been shown. The absorbance (A) was calculated as 1 - transmittance (T) - reflectance (R). T and R were obtained from simulations for the slot antennas without external magnetic field. **(b)** The absorption coefficient spectra of each sample have been shown. The absorption coefficients (α) are calculated by $\alpha = 2.302 \times \mathrm{A} \,/\, (100\ nm)$.

S6. Effective medium analysis for slot antenna array

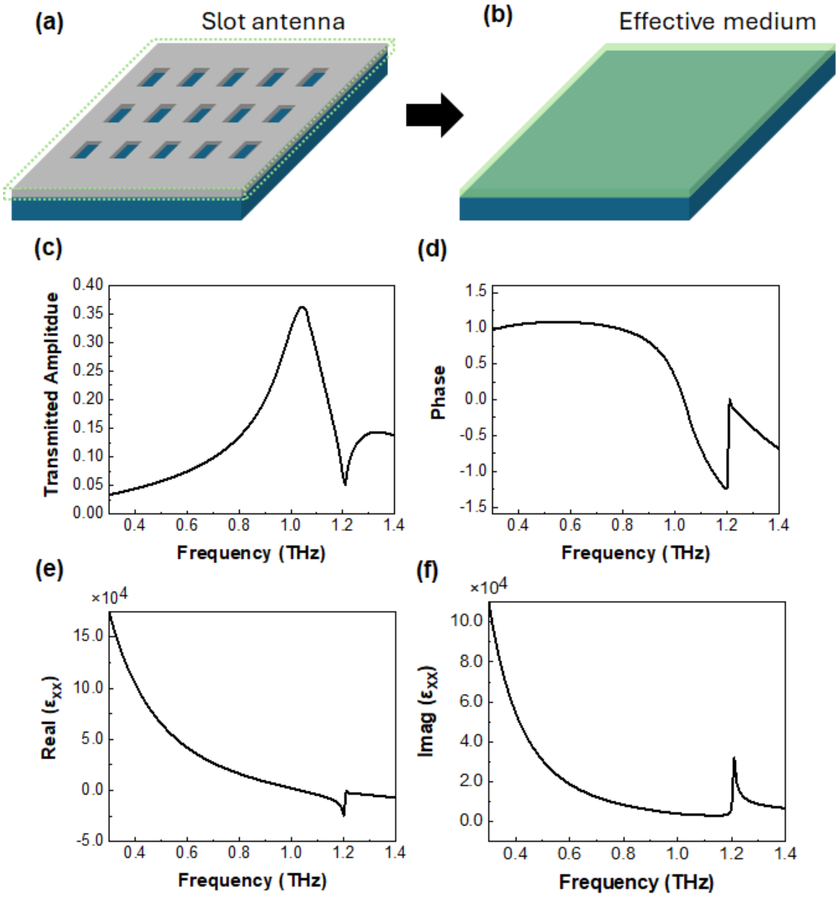

Figure S6. Using the effective medium theory, (a) the iron slot antennas without magnetic field is converted to (b) the effective medium. As a thin film, the effective medium has the same thickness of 100 nm as the slot antenna sample. (c) The transmitted amplitude and (d) phase information can be obtained from the simulations with the 1 μm-width slot antennas. Using Fresnel's equation, the effective dielectric constant of the effective medium can be extracted based on transmitted amplitude and phase information [6]. The extracted (e) real part and (f) imaginary part of the effective dielectric constant are shown.

References

[1] J. Neu and C. A. Schmuttenmaer, "Tutorial: An introduction to terahertz time domain spectroscopy (THz-TDS)," *J. Appl. Phys.,* vol. 124, no. 23, 2018, https://doi.org/10.1063/1.5047659

[2] P. Duhamel and M. Vetterli, "Fast Fourier-Transforms - a Tutorial Review and a State-of-the-Art," *Signal Process.,* vol. 19, no. 4, pp. 259-299, 1990, https://doi.org/10.1016/0165-1684(90)90158-U

[3] C. M. Morris, R. V. Aguilar, A. V. Stier, and N. P. Armitage, "Polarization modulation time-domain terahertz polarimetry," *Opt. Express,* vol. 20, no. 11, pp. 12303-12317, 2012, https://doi.org/10.1364/Oe.20.012303

[4] Y. F. Chen, "Nanofabrication by electron beam lithography and its applications: A review," *Microelectron. Eng.*, vol. 135, pp. 57-72, 2015, https://doi.org/10.1016/j.mee.2015.02.042

[5] A. L. Bogdanov, J. Lapointe, and J. H. Schmid, "Electron-beam lithography for photonic waveguide fabrication: Measurement of the effect of field stitching errors on optical performance and evaluation of a new compensation method," *J. Vac. Sci. Technol. B,* vol. 30, no. 3, 2012, https://doi.org/10.1116/1.3700439

[6] H. T. Lee *et al.*, "Measuring Complex Refractive Indices of a Nanometer-Thick Superconducting Film Using Terahertz Time-Domain Spectroscopy with a 10 Femtoseconds Pulse Laser," *Crystals,* vol. 11, no. 6, 2021, https://doi.org/10.3390/cryst11060651
